# Strong Metal‐Support Interaction Driven Janus Reconstruction in PtRu Alloys for Low‐Temperature Hydrogen Storage

**DOI:** 10.1002/advs.77432

**Published:** 2026-08-27

**Authors:** Xingyu Zhang, Haiqiang Bai, Wenbin Li, You Wang, Yunhua Xu, Sai Zhang

**Affiliations:** ^1^ College of New Energy Yulin University Yulin China; ^2^ School of Material Science and Engineering Xi'an University of Technology Xi'an China; ^3^ School of Chemistry and Chemical Engineering Northwestern Polytechnical University Xi'an China

**Keywords:** janus reconstruction, low‐temperature hydrogen storage, PtRu alloys, strong metal‐support interaction (SMSI), toluene hydrogenation

## Abstract

Janus nanostructures enable the spatial decoupling distinct catalytic functions, yet their controlled synthesis remains challenging, as bimetallic systems thermodynamically favor alloy formation. Herein, we demonstrate that strong metal‐support interaction (SMSI) can drive a transformation from alloy to Janus nanoparticles. Using a PtRu alloy on CeO_2_ as a model system, the inherently stronger interaction of Ru with CeO_2_ directs Ru to migrate toward the metal‐support interface during high‐temperature reduction, thereby forcing Pt to segregate into adjacent, Pt‐enriched domains. This SMSI‐driven reconstruction spontaneously generates atomically intimate Janus nanoparticles, spatially partitioned Pt‐rich and Ru‐rich regions for H_2_ dissociation and toluene adsorption, respectively. Facilitated by hydrogen spillover across the seamless interface, this spatial organization enables low‐temperature hydrogen storage via toluene hydrogenation, achieving a turnover frequency of 10 906 h^−1^ at 50°C. This value represents a 6.7‐fold enhancement over the alloyed nanoparticles and surpasses state‐of‐the‐art catalysts that operate above 100°C. This study establishes SMSI as a thermodynamic lever for creating multiple active sites within a single nanoparticle, offering a rational and scalable pathway to advanced catalysts for energy storage and conversion.

## Graphical abstract

Strong metal‐support interactions (SMSI) drive the spontaneous segregation of RuPt alloy into Janus nanoparticles on CeO_2_, spatially partitioning Pt‐rich domains for H_2_ dissociation and Ru‐rich domains for toluene adsorption. This Janus nanocatalysts yield a turnover frequency of 10 906 h^−1^ for toluene hydrogenation at 50 °C, which is 6.7 times that of the alloyed counterpart and higher than reported values for state‐of‐the‐art catalysts operating above 100 °C.

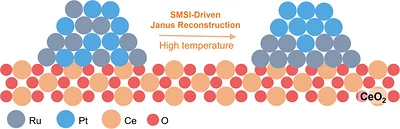

## 1 Introduction

Rational design of catalytic nanostructures is pivotal for advancing heterogeneous catalysis toward sustainable energy and chemical processes [1, 2, 3]. A persistent challenge lies in efficiently catalyzing multi‐step reactions involving reactants with distinct chemical properties, for instance, the simultaneous activation of non‐polar H_2_ and aromatic molecules [4, 5, 6]. Janus nanoparticles have emerged as a transformative platform in this context [7, 8, 9]. Featuring two compositionally and functionally distinct domains connected by an atomically intimate interface (as characterized by electron microscopy with EDX or EELS elemental mapping/line scanning), these catalysts circumvent the electronic and geometric averaging that limits multifunctional synergy in monometals or alloys [10, 11, 12]. This spatial partitioning is particularly powerful for multi‐step transformations: one domain activates one reactant while its counterpart handles another, with intermediates shuttled across their interface [11, 13, 14]. A compelling application is hydrogen storage via liquid organic hydrogen carriers (LOHCs), particularly the hydrogenation of toluene to methylcyclohexane [15, 16, 17, 18, 19, 20, 21, 22]. This reaction is critical for reversible H_2_ storage owing to its high gravimetric capacity (6.1 wt.% H_2_), yet it demands both efficient H_2_ dissociation and effective toluene activation without mutual interference.

Despite their conceptual appeal, the controlled synthesis of well‐defined Janus nanoparticles remains a challenge [7, 8]. Thermodynamically, bimetallic systems favor the formation of solid‐solution alloys to minimize interfacial free energy [12, 23], rendering the creation and maintenance of asymmetric, phase‐separated structures inherently difficult for unsupported Janus nanoparticles, particularly for the nanoscale particles (Figure 1a) [23]. Immobilizing such Janus nanoparticles onto a support surface offers a viable strategy to stabilize them (Figure 1a). However, existing synthetic approaches, including seed‐mediated growth, self‐assembly, and microemulsion methods, often suffer from limited reproducibility, heterogeneous particle populations, and insufficient control over domain size, composition, and interfacial integrity [24, 25, 26, 27]. These shortcomings obscure definitive structure‐performance relationships and hinder the practical applications of Janus nanocatalysts, particularly where precise interface engineering dictates catalytic synergy.

**FIGURE 1:**
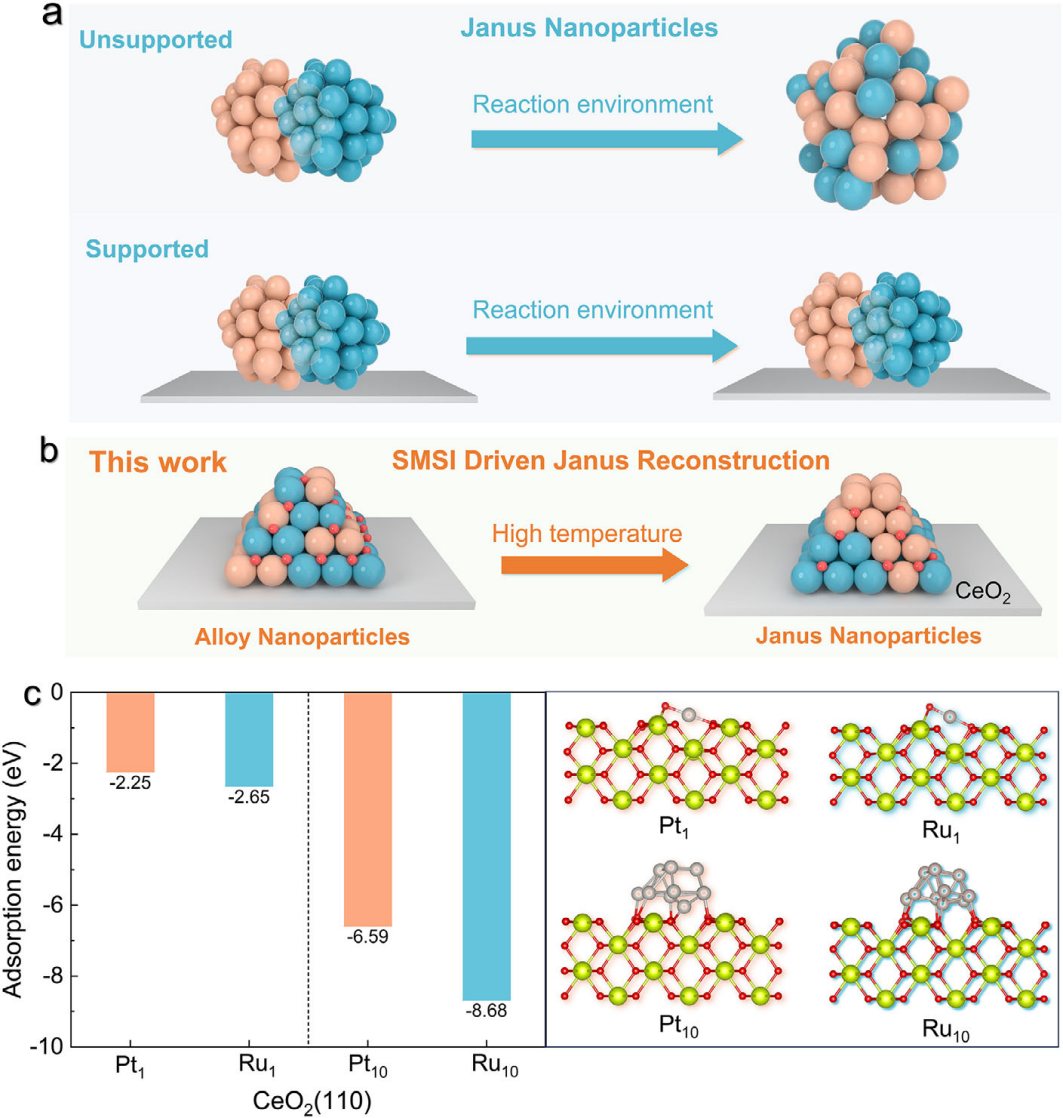
SMSI‐driven Janus reconstruction of PtRu alloy nanoparticles and theoretical validation. (a) Schematic evolution of Janus nanoparticles in unsupported and supported states under catalytic conditions. (b) Schematic of the SMSI‐induced structural reconstruction of PtRu alloy nanoparticles into Janus nanoparticles on CeO_2_ support. (c) DFT calculated adsorption energies of single Pt/Ru atoms (Pt_1_, Ru_1_) and 10‐atom Pt/Ru clusters (Pt_10_, Ru_10_) on the CeO_2_(110) surface, with corresponding structural models.

Herein, we report a thermodynamically driven strategy to construct Janus nanostructures on CeO_2_ by leveraging differential strong metal‐support interactions (SMSI). As illustrated in Figure 1b, during high‐temperature reduction, the stronger interaction between Ru and CeO_2_ preferentially anchors Ru at the metal‐support interface, while simultaneously driving the segregation of the more weakly interacting Pt to from atomically intimate Janus nanostructure with spatially partitioned Pt‐rich and Ru‐rich domains. This configuration decouples the complementary functions: Pt‐rich regions serve as highly active sites for H_2_ dissociation, whereas Ru‐rich domains facilitate toluene adsorption and activation. Facilitated by the seamless interface, ^*^H spillover from Pt to Ru sites enables an exceptional activity for low‐temperature toluene hydrogenation, achieving a remarkable turnover frequency (TOF) of 10 906 h^−1^ at 50°C, a value that significantly surpasses state‐of‐the‐art catalysts typically operating above 100°C.

## 2 Results and Discussion

### 2.1 Theoretical Analysis of SMSI Driving Janus Reconstruction

To provide a theoretical foundation for the SMSI‐driven Janus reconstruction, we performed density functional theory (DFT) calculations. CeO_2_ was chosen as the support, because it is a typical oxide for studying strong metal‐support interaction [28, 29, 30], and the SMSI between Ru and CeO_2_ has been well established in the literature [31, 32, 33]. Among the various facets, we selected the CeO_2_(110) surface as the model support. Because its oxygen vacancy formation energy is moderate, neither too high to be inaccessible nor too low to be unstable. This characteristic renders the CeO_2_(110) facet offers abundant active sites including oxygen vacancies and Ce^3+^ sites that are known to mediate metal‐support interactions [34, 35, 36, 37, 38]. To investigate the binding strengths of Pt and Ru with the CeO_2_(110) surface, we compared the adsorption energies of single metal atoms (M_1_) and ten‐atom metal clusters (M_10_), which allows us to evaluate the size‐dependent metal‐support interaction at both atomic and nanoscale levels.

The calculations reveal a pronounced difference in binding strength. As shown in Figure 1c and Figure S1, the adsorption energy of a Ru_1_ atom was calculated to be −2.65 eV, which is stronger than that of a Pt_1_ atom (−2.25 eV), indicating a more favorable and intense interaction between Ru and the CeO_2_ support. Crucially, this discrepancy in Ru‐CeO_2_ interaction is not only maintained but amplified with increasing metal cluster size. When the model was scaled to a 10‐atom cluster, the Ru_10_ cluster (−8.68 eV) exhibits a stronger interaction with CeO_2_(110) compared to Pt_10_ cluster (−6.59 eV, Figure 1c and Figure S2). This trend confirms that the driving force for Janus reconstruction is not merely an atomic‐scale phenomenon but is enhanced at the nanoscale.

Collectively, these DFT results provide a theoretical foundation for our proposed Janus reconstruction mechanism. The stronger metal‐support interaction between Ru and CeO_2_, compared to Pt, creates a definitive thermodynamic driving force (Figure 1b). We propose that during catalyst activation (e.g., high‐temperature reduction), this force will trigger a structural reconstruction: Ru atoms are preferentially pulled and anchored at the metal‐support interface, which in turn expels the more mobile Pt atoms, forcing their segregation into distinct, Pt‐enriched domains adjacent to the Ru‐anchored interface. Therefore, the initial PtRu alloy is reconstructed into a Janus nanostructure featuring intimately spaced Pt‐rich and Ru‐rich domains. This theoretical prediction of a differential‐SMSI‐induced Janus reconstruction directly rationalizes the spontaneous creation of the spatially decoupled active sites, which is the cornerstone of the synergistic catalysis we aim to achieve.

### 2.2 Realizing Janus Reconstruction of PtRu on CeO_2_

Guided by the DFT predictions, we experimentally realized the SMSI‐driven Janus reconstruction via controlled high‐temperature reduction. CeO_2_ nanorods were prepared as the support via a hydrothermal method. x‐ray diffraction (XRD) patterns confirmed the fluorite structure of the as‐synthesized CeO_2_ nanorods (Figure S3a). Transmission electron microscopy (TEM) images revealed their characteristic rod‐like morphology (Figure S3b). High‐resolution TEM image, coupled with corresponding fast Fourier transform (FFT) analysis, resolved lattice fringe spacings of 0.19 and 0.31 nm, corresponding to the (110) and (111) planes of CeO_2_, respectively (Figure S3c,d). This crystallographic feature is consistent with the well‐documented growth orientation of CeO_2_ nanorods, which grow preferentially along the [110] direction, exposing the (110) facets on the side surfaces and terminating with (111) facets at the tips [38, 39].

To trigger the proposed reconstruction, we deposited PtRu precursors on the CeO_2_ supports via the impregnation method, and the resulting samples were subsequently reduced at 200°C, 350°C, and 500°C under a H_2_/Ar (5 *vol*.%) atmosphere. Notably, the CeO_2_ supports retained its phase and structural integrity (Figure S4). We hypothesized that these temperatures serve as a kinetic switch: at 200°C, alloy formation might dominate; at 500°C, the differential SMSI drives complete Janus reconstruction; and at 350°C, an intermediate state is captured. The resulting catalysts were denoted as PtRu/CeO_2_‐200, PtRu/CeO_2_‐350, and PtRu/CeO_2_‐500, respectively. Inductively coupled plasma optical emission spectrometry (ICP‐OES) tests determined the actual Pt and Ru loadings were comparable and summarized in Table 1, ruling out significant metal loss during reduction.

**TABLE 1:** Summary of the actual metal loading.

Entry	Catalysts	Pt loading (wt.%)	Ru loading (1 wt.%)
1	PtRu/CeO_2_‐200	1.0	1.9
2	PtRu/CeO_2_‐350	1.1	2.1
3	PtRu/CeO_2_‐500	1.0	1.9
4	Pt/CeO_2_‐500	3.1	0
5	Ru/CeO_2_‐500	0	2.9

The SMSI‐driven reconstruction was directly visualized at the nanoscale using aberration‐corrected high‐angle annular dark‐field scanning transmission electron microscopy (HAADF‐STEM) coupled with energy‐dispersive x‐ray (EDX) spectroscopy. Notably, the metal nanoparticles were predominantly distributed on the side surfaces of the CeO_2_ nanorods (Figure 2a–d). This observation confirms that the primary interaction occurs with the exposed CeO_2_(110) facets, which is consistent with our DFT model. For PtRu/CeO_2_‐200, HAADF‐STEM image revealed well‐defined nanoparticles uniformly dispersed on CeO_2_ (Figure 2a), with an average size of 0.9 ± 0.1 (Figure S5). EDX line‐scan profiles across individual nanoparticles indicated a close overlap of Pt and Ru signals, confirming a alloy structure (Figure 2b). In contrast, PtRu/CeO_2_‐500 exhibited a clear structural transformation. While the particle size increased to 1.2 ± 0.1 nm (Figure 2c and Figure S6), EDX line‐scan analysis revealed a distinct spatial decoupling of the elemental signals. Specifically, the Ru signal was consistently enriched at the metal‐support interface, whereas Pt dominated the particle interior and the surface away from CeO_2_ (Figure 2d). These observations provide direct nanoscale evidence that high‐temperature reduction drives the SMSI‐induced reconstruction of PtRu nanoparticles, inducing a thermodynamically favored segregation of Ru toward the CeO_2_ interface.

**FIGURE 2:**
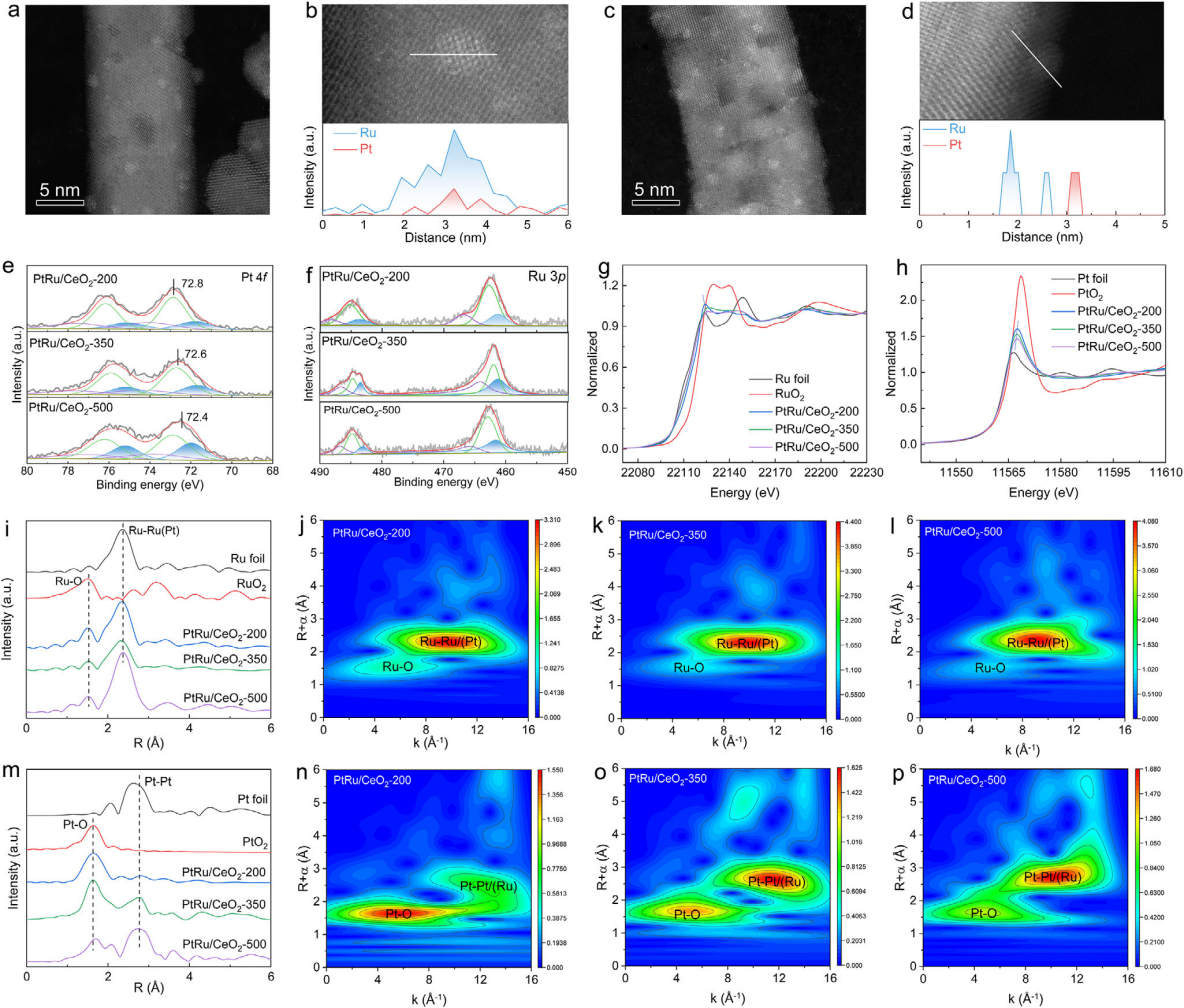
Nanoscale and electronic evidence for SMSI‐driven PtRu Janus reconstruction. (a) HAADF‐STEM image and (b) EDX line‐scan elemental profiles of PtRu/CeO_2_‐200. (c) HAADF‐STEM image and (d) EDX line‐scan elemental profiles of PtRu/CeO_2_‐500. XPS spectra of (e) Pt 4*f* and (f) Ru 3*p* for PtRu/CeO_2_‐200, PtRu/CeO_2_‐350 and PtRu/CeO_2_‐500. XANES spectra at the (g) Ru K‐edge and (h) Pt L_3_‐edge for PtRu/CeO_2_‐200, PtRu/CeO_2_‐350 and PtRu/CeO_2_‐500. FT extended EXAFS analysis at the (i–l) Ru K‐edge and (m–p) Pt L_3_ edge for PtRu/CeO_2_‐200, PtRu/CeO_2_‐350 and PtRu/CeO_2_‐500.

X‐ray photoelectron spectroscopy (XPS) provided complementary electronic evidence for the Janus reconstruction and its interfacial origin. Although the PtRu/CeO_2_‐T catalysts were reduced at different temperatures, the surface Ce^3+^ and associated oxygen vacancies of the CeO_2_ supports remained well preserved. Specifically, the Ce^3+^ fractions were determined to be 30.9%, 30.1% and 30.8% for PtRu/CeO_2_‐200, PtRu/CeO_2_‐350 and PtRu/CeO_2_‐500, respectively (see Supplementary Materials for detailed calculation, Figure S7a). While the corresponding fractions of oxygen vacancy‐associated species (Ce^3+^‐O) were 69.1%, 69.9% and 66.2%, respectively (Figure S7b). In contrast, the electronic properties of supported metals exhibited a temperature‐dependent divergence. As shown in Figure 2e and Table S1, with increasing reduction temperature, the Pt 4*f* spectra revealed a gradual decrease in binding energy form 72.8 to 72.4 eV, along with a progressive increase in the Pt° fraction. Conversely, the Ru 3*p* spectra showed a largely unchanged Ru° fraction for PtRu/CeO_2_‐T (Figure 2f and Table S2). This dichotomy reflects the differential metal‐support interaction imposed by the reconstruction: Ru atoms, anchored at the interface through strong SMSI, retain their electron interaction with the support; Pt atoms, expelled into enriched domains away from the interface, experience diminished interfacial perturbation and thus undergo more complete reduction to the metallic state. The compositional segregation visualized by STEM‐EDX is therefore mirrored by a corresponding spatial differentiation in electronic structure, further validating the SMSI‐driven Janus nanoparticles.

To gain further atomic‐level insight into the Janus reconstruction, we employed synchrotron‐based x‐ray absorption spectroscopy. X‐ray absorption near‐edge structure (XANES) spectra confirmed the predominantly metallic states of both Ru and Pt across all catalysts. However, a detailed comparison revealed a temperature‐dependent divergence in their electronic environment. The Ru K‐edge spectra exhibited a subtle but consistent shift toward higher energy form PtRu/CeO_2_‐200 to PtRu/CeO_2_‐350 and then to PtRu/CeO_2_‐500 (Figure 2g), approaching the profile of RuO_2_. This trend indicates a gradual increase in Ru‐O bond character, consistent with Ru strengthening its interaction with CeO_2_ support. In contrast, the Pt L3‐edge spectra shifted progressively toward the Pt foil with enhancing reduction temperature (Figure 2h). This trend signals a rise in the metallic Pt‐Pt character, reflecting the aggregation of Pt into enriched domains.

This electronic structure interpretation was further corroborated by the extended x‐ray absorption fine structure (EXAFS) analysis, which probed the local coordination geometry. A simultaneous fitting analysis of both Pt and Ru edge, accounting for homometallic (Pt‐Pt and Ru‐Ru) and heterometallic (Pt‐Ru and Ru‐Pt) interactions, was performed. Fourier‐transformed EXAFS at the Ru K‐edge showed minimal change in the Ru‐O and Ru‐Ru(Pt) scattering paths across the catalyst series (Figure 2i–l, Figure S8 and Table S2), indicating stable Ru coordination. Conversely, the Pt L3‐edge EXAFS exhibited a marked enhancement in the Pt‐Pt scattering path amplitude for PtRu/CeO_2_‐500 (Figure 2m). Quantitative fitting confirmed an increase in the Pt‐Pt/Ru coordination number alongside a decrease in Pt–O coordination (Figure 2n–p, Figure S9 and Table S2), unambiguously demonstrating Pt segregation and domain coalescence.

Collectively, this multi‐faceted spectroscopic and microscopic investigation provides definitive evidence for the SMSI‐driven Janus reconstruction. Notably, this Janus nanostructure is achieved through a simple thermal treatment on conventional supported alloy nanoparticles, no elaborate colloidal synthesis or seeded growth is required. The key lies in harnessing the support itself as an active component: the strong and preferential interaction between Ru and CeO_2_ serves as an in situ thermodynamic anchor, drawing Ru toward the interface and simultaneously expelling Pt into adjacent domains. This SMSI‐mediated reconstruction circumvents a long‐standing challenge in Janus nanoparticle synthesis, the thermodynamic tendency of isolated, unsupported Janus particles to alloy and minimize their interfacial free energy. By anchoring the Janus nanoparticles onto a reducible support, the reconstructed configuration becomes kinetically trapped and thermodynamically stabilized under reaction conditions.

### 2.3 Janus Nanoparticles for Toluene Hydrogenation

The SMSI‐driven Janus reconstruction yields a pronounced enhancement in catalytic hydrogenation performance. We evaluated the hydrogenation of toluene to methylcyclohexane over PtRu/CeO_2_‐200, PtRu/CeO_2_‐350, and PtRu/CeO_2_‐500 under solvent‐free conditions at 50°C and 1 MPa H_2_. The Pt:Ru ratio was optimized as 1:2, which afforded the highest toluene conversion (Figure S10). Unless otherwise specified, all PtRu/CeO_2_‐T catalysts maintained this optimal ratio. Toluene conversion increased markedly with enhanced reduction temperature, correlating with the progressive formation of the Janus nanoparticles. The PtRu/CeO_2_‐200 catalysts, which retains an alloyed PtRu structure, gave a conversion of 27%; and this significantly rose to 70% for PtRu/CeO_2_‐350. Notably, the PtRu/CeO_2_‐500 catalyst with complete Ru segregation toward the CeO_2_ interface achieved the highest toluene conversion of 93% (Figure 3a).

**FIGURE 3:**
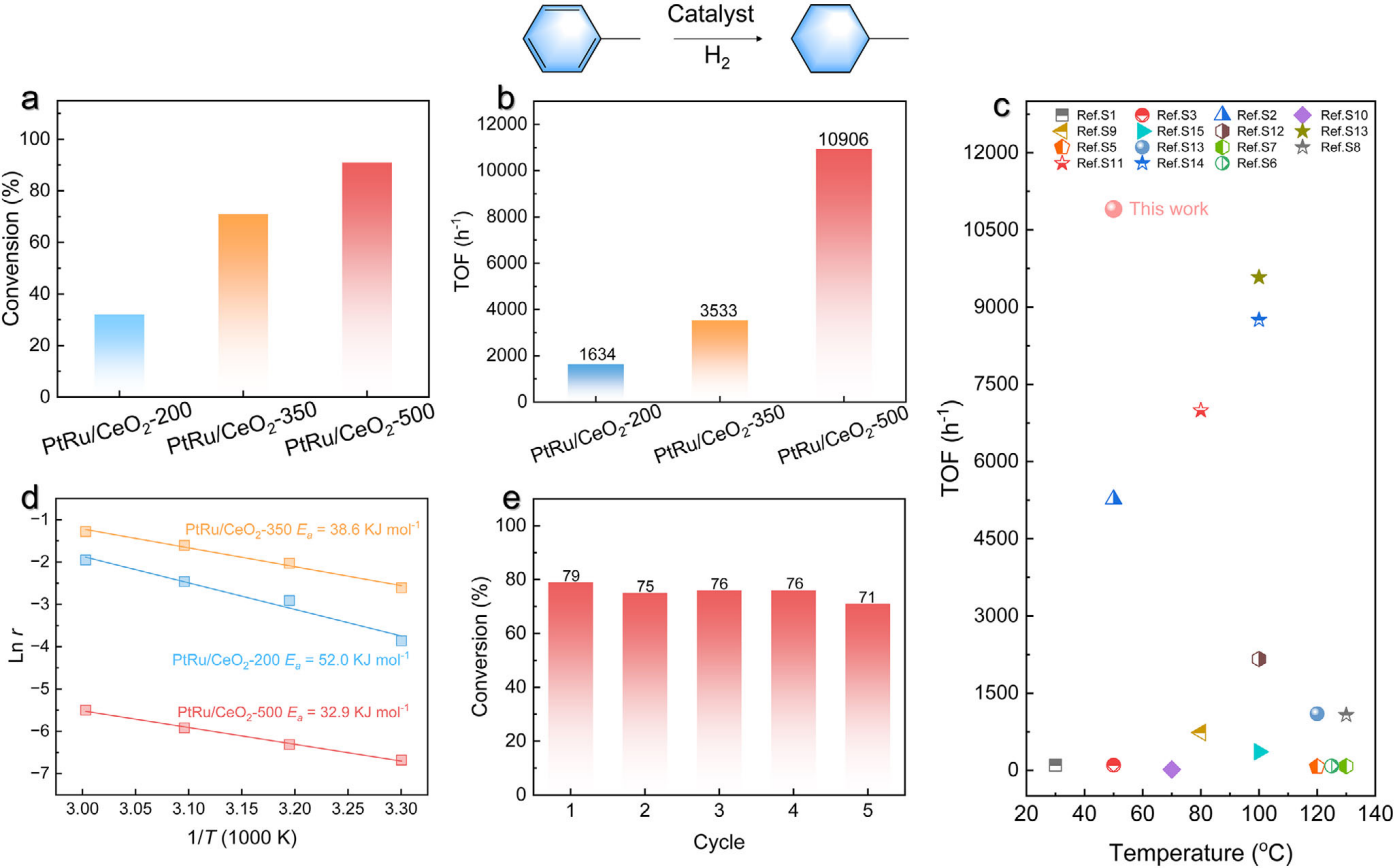
Catalytic performance. (a) Toluene conversion over PtRu/CeO_2_‐200, PtRu/CeO_2_‐350, and PtRu/CeO_2_‐500 for toluene hydrogenation. Reaction conditions: catalysts (10 mg), toluene (1 mL), 50 °C, 1 MPa H_2_ and 2 h. (b) TOF values. *Note*: TOF calculated for surface metal sites under kinetic control, toluene conversion <30%. (c) Comparison of TOF for PtRu/CeO_2_‐500 against state‐of‐the‐art toluene hydrogenation catalysts (details in Supplementary Information). (d) Arrhenius plots and apparent *E_a_* for PtRu/CeO_2_‐T catalysts. (e) Cycling stability of PtRu/CeO_2_‐500 over five consecutive runs. Reaction conditions: PtRu/CeO_2_‐500 (10 mg), toluene (1 mL), 50 °C, 1 MPa H_2_ and 2 h.

This trend is even more evident in the turnover frequency (TOF), which normalizes the intrinsic activity per surface metal site (Figure 3b). PtRu/CeO_2_‐200 showed the lowest TOF of 1634 h^−1^, which increased to 3533 h^−1^ for PtRu/CeO_2_‐350. Remarkably, the PtRu/CeO_2_‐500 catalyst with Janus PtRu nanoparticles achieved a TOF of 10 906 h^−1^, an approximately 12‐fold enhancement relative to PtRu/CeO_2_‐200 with the PtRu alloys. To contextualize this performance, we benchmarked PtRu/CeO_2_‐500 against state‐of‐the‐art toluene hydrogenation catalysts reported in the literature. Notably, its TOF at 50°C substantially exceeds those of most reference systems, which typically operate above 100°C and deliver TOFs below 2000 h^−1^ (Figure 3c and Table S3).

Kinetic analysis via Arrhenius plots further rationalizes this superior activity. PtRu/CeO_2_‐500 exhibited a linear increase in toluene conversion with reaction time (Figure S11a), characteristic of zero‐order kinetics and confirming negligible mass transfer limitations [40, 41]. Across the temperature range of 30°C –60°C, all PtRu/CeO_2_‐T catalysts displayed a consistent activity trend (Figure S11b). Arrhenius analysis yielded the apparent activation energy (*E_a_*) for the toluene hydrogenation over each PtRu/CeO_2_‐T catalyst (Figure 3d). Specifically, the PtRu/CeO_2_‐500 catalyst exhibits the lowest *E_a_* of 32.9 kJ mol^−1^, compared to 52.0 kJ mol^−1^ for PtRu/CeO_2_‐200 and 38.6 kJ mol^−1^ for PtRu/CeO_2_‐350. This substantial reduction in activation energy reflects the synergistic cooperation between the spatially decoupled Pt and Ru domains enabled by the Janus reconstruction.

The stability of the PtRu/CeO_2_‐500 catalyst was evaluated through consecutive toluene hydrogenation cycles (Figure 3e). After five cycles, the toluene conversion remained >70% (initial ∼79%), indicating only minimal deactivation. HAADF‐STEM image of the spent PtRu/CeO_2_‐500 catalyst revealed well‐dispersed metal nanoparticles on the CeO_2_ surface (Figure S12a), with an average size of 1.2 ± 0.2 nm (Figure S12b), comparable to that of the fresh catalyst (1.2 ± 0.1 nm). XRD analysis showed no observable changes in the diffraction pattern relative to the fresh sample, with no additional peaks attributable to metallic Pt or Ru (Figure S13a). XPS analysis further confirmed that the binding energies of Ru 3*p* (Figure S13b) and Pt 4*f* (Figure S13c) remained essentially unchanged, with Pt^0^ and Ru° fractions comparable to those of the fresh catalyst (Tables S1 and S2). These results confirm that both the geometrical and electronic structures of the Janus nanoparticles are well preserved under repeated reaction conditions. This robust recyclability, combined with its record‐high TOF and low *E_a_*, underscores the pivotal role of SMSI‐induced Janus reconstruction in enabling superior toluene hydrogenation performance. Overall, PtRu/CeO_2_‐500 outperforms both alloy PtRu materials and state‐of‐the‐art catalysts for this reaction.

### 2.4 The Critical Role of Janus Nanostructure

The exceptional performance originates from the distinct and complementary catalytic functions of the spatially separated Pt and Ru domains, as rationalized by *d*‐band theory. According to *d*‐band center theory [42, 43, 44], the position of the *d*‐band center relative to the Fermi level serves as a fundamental descriptor for adsorption strength of reactants and intermediates on transition‐metal surfaces. Typically, an up‐shifted *d*‐band center strengthens the adsorption of key intermediates, which can enhance specific catalytic steps. While a down‐shifted center weakens adsorption and may optimize the overall activity by balancing bond formation and product desorption.

To establish appropriate surface models for DFT calculations, we constructed models that faithfully represent the key structural features of our catalysts while remaining computationally tractable. For the pure metals, we adopted the most stable and commonly exposed facets: Pt(111) for face‐centered cubic (FCC) Pt and Ru(0001) for hexagonal close‐packed (HCP) Ru. For the Janus nanoparticles, we constructed a Ru(0001)/Pt(111) interface to mimic the spatially separated Pt‐rich and Ru‐rich domains observed in PtRu/CeO_2_‐500. For the alloy nanoparticles, the HAADF‐STEM image of PtRu/CeO_2_‐200 revealed a lattice spacing of 0.22 nm (Figure S14), characteristic of the Pt(111) facet; we therefore adopted a PtRu(111) alloy surface model to represent the homogeneous alloy structure.

DFT calculations reveal that Pt(111) exhibits a lower *d*‐band center of −2.00 eV compared to Ru(0001) of −1.51 eV (Figure 4a). This distinct difference in *d*‐band center endows Pt and Ru with markedly different adsorption and catalytic properties. In contrast, when the Ru atom is alloyed in Pt(111), the resulting PtRu(111) alloy exhibits the uniform *d*‐band center averaging −1.73 eV across the surface (Figure 4b). Importantly, in such an alloyed configuration, the electronic structure becomes homogenized, with the *d*‐band centers of the upper and lower atomic layers converging to the same value. For reactions requiring simultaneous activation of chemically distinct molecules, such as H_2_ and toluene, this electronic uniformity is suboptimal, as it compromises the ability to efficiently activate both reactants. This explains why the alloyed structure in PtRu/CeO_2_‐200 exhibits limited catalytic performance compared to the Janus PtRu nanoparticles in PtRu/CeO_2_‐500 for toluene hydrogenation.

**FIGURE 4:**
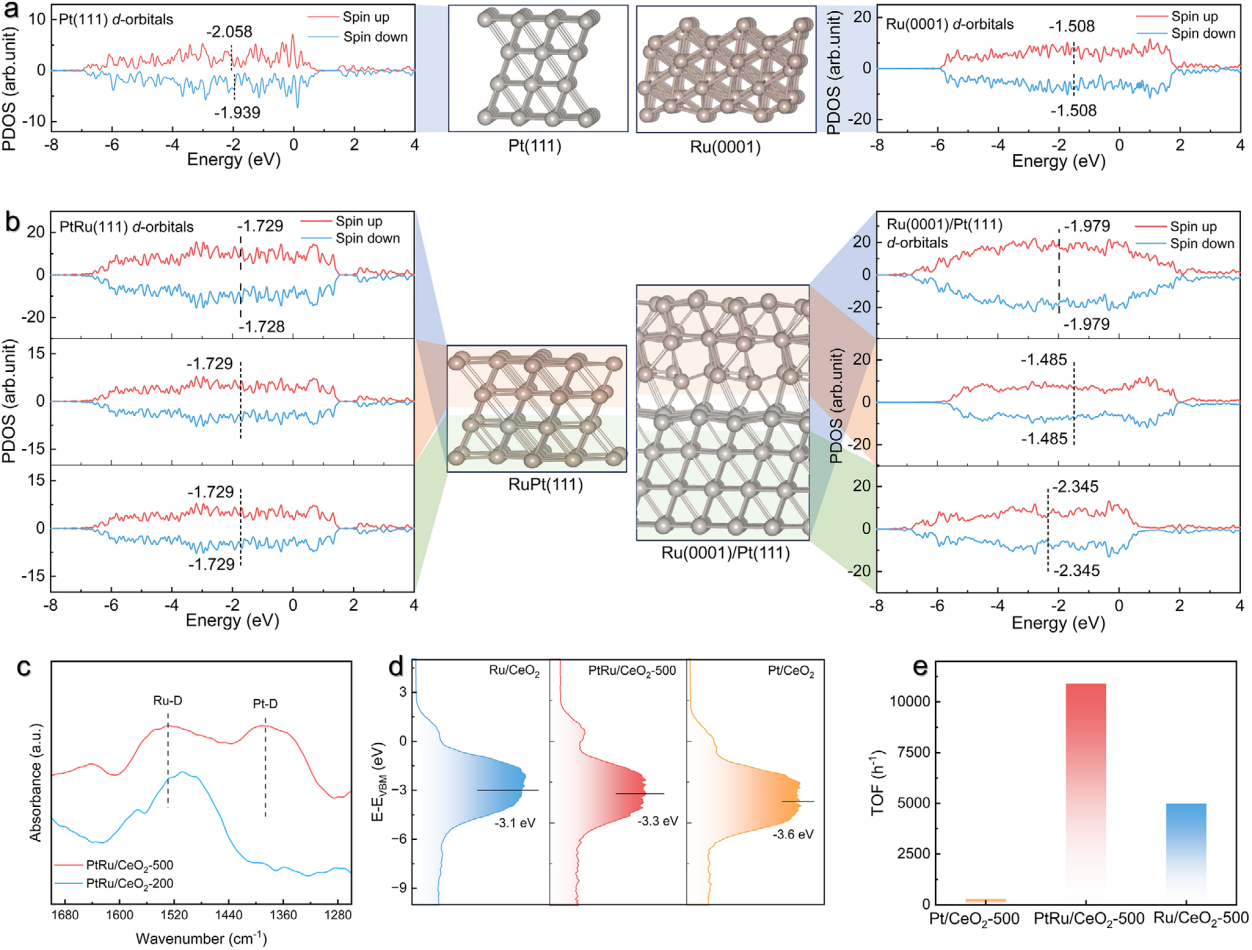
Electronic structure and functional specialization of Janus PtRu nanostructure. (a) Projected density of states (PDOS) of d‐orbitals for Pt(111) and Ru(111) surfaces. (b) PDOS of d‐orbitals for PtRu(111) alloy and Janus Pt(111)Ru(111) surfaces; Vertical dashed lines denote calculated d‐band centers. (c) In situ DRIFT spectra of PtRu/CeO_2_‐200 and PtRu/CeO_2_‐500 after D_2_ exposure. (d) d‐band centers of the PtRu/CeO_2_‐200, PtRu/CeO_2_‐350 and PtRu/CeO_2_‐500 catalysts. (e) TOF comparison of PtRu/CeO_2_‐500 with monometallic Pt/CeO_2_‐500 and Ru/CeO_2_‐500. **Reaction conditions**: catalysts (5 mg), toluene (1 mL), 50 °C, 1 MPa H_2_ and 2 h.

In the Janus Ru(0001)/Pt(111) structure, interaction between the two metals shifts the overall d‐band center of the composite system to −1.98 eV (Figure 4b). Crucially, unlike the alloyed case, the local d‐band centers within the Ru and Pt domains remain largely preserved, at −1.49 and −2.35 eV, respectively (Figure 4b). This retention of region‐specific electronic structures enables Ru and Pt in the Janus configuration to maintain their intrinsic chemical properties, Ru favoring strong adsorption and hydrogenation of toluene, while Pt excels in H_2_ dissociation. Thus, the Janus nanostructure successfully decouples and preserves the complementary functions of each metal, leading to the dramatically enhanced catalytic activity observed for toluene hydrogenation.

To further probe the distinct electronic properties within the Janus nanostructure, we performed in situ diffuse reflectance infrared Fourier transform spectroscopy (DRIFTS) to monitor the metal‐D vibrational bands. After cleaning the PtRu/CeO_2_‐200 and PtRu/CeO_2_‐500 surface in Ar flow (30 mL min^−1^) at 30°C for 30 min, the D_2_/Ar flow (10 *vol*.%, 30 mL min^−1^) was introduced. For PtRu/CeO_2_‐200, a single characteristic peak at 1505 cm^−1^ reflects the averaged and uniform electronic properties (Figure 4c) [45, 46]. In contrast, PtRu/CeO_2_‐500 exhibits two distinct peaks at 1527 and 1386 cm^−1^, corresponding to Ru‐D and Pt‐D bands, respectively (Figure 4c). This clear spectroscopic separation directly confirms the significantly different electronic environments of Ru and Pt in the PtRu/CeO_2_ catalysts with Janus structure.

For comparison, the monometallic Pt/CeO_2_‐500 and Ru/CeO_2_‐500 catalysts were prepared following identical procedure as PtRu/CeO_2_‐500. ICP‐OES analysis determined the Pt loading of Pt/CeO_2_‐500 to be 3.1 wt.% and the Ru loading of Ru/CeO_2_‐500 to be 2.9 wt.% (Table 1). HAADF‐STEM images confirmed comparable particle sizes for Pt (1.2 ± 0.2 nm) and Ru (1.2 ± 0.1 nm) on Pt/CeO_2_‐500 (Figure S15) and Ru/CeO_2_‐500 (Figure S16), respectively, enabling a fair assessment of intrinsic activities. As expected from DFT predictions, the *d*‐band center of Ru/CeO_2_‐500 was significantly higher than that of Pt/CeO_2_‐200, as determined by high‐resolution valence‐band XPS (Pt 4*f* and Ru 3*p*) referenced to the valence band maximum (Figure 4d). However, because XPS measures a statistical average across the ensemble, the PtRu/CeO_2_‐500 catalyst exhibited only a single, centralized *d*‐band center, precluding the direct deconvolution of the individual Pt and Ru contributions (Figure 4d).

To further validate the electronic origin of the Janus reconstruction, we compared the XPS spectra of the monometallic references with those of the alloy and Janus catalysts. The Ru 3*p* spectra of Ru/CeO_2_‐500, PtRu/CeO_2_‐200, and PtRu/CeO_2_‐500 exhibited comparable binding energies (Figure S17a), indicating that the Ru electronic structure is primarily governed by the strong metal‐support interaction with CeO_2_. In contrast, Pt 4*f* analysis revealed that Pt/CeO_2_‐500 exhibited significantly lower binding energy and higher Pt° fraction than PtRu/CeO_2_‐200 (Figure S17b), confirming that Pt is more readily reduced alone. In the alloy, the Pt binding energy shifted positively due to the electronic influence of Ru, particularly given that the Ru:Pt ratio is 2:1. Importantly, the Janus PtRu/CeO_2_‐500 catalyst exhibited the Pt 4*f* binding energy of 72.4 eV closer to that of pure Pt/CeO_2_‐500 (72.1 eV), along with enhanced Pt° fraction (Table S1). These features indicate that upon Janus reconstruction, Pt atoms are expelled from the alloy environment into Pt‐enriched domains, where they recover electronic characteristics more similar to those of monometallic Pt.

Furthermore, the functional benefits of this electronic differentiation are reflected in catalytic performance. For toluene hydrogenation, the Janus nanostructure in PtRu/CeO_2_‐500 resulted in a pronounced enhancement performance compared to the monometallic Pt/CeO_2_‐500 and Ru/CeO_2_‐500 catalysts (Figure S18). Under identical reaction conditions, the TOF of PtRu/CeO_2_‐500 (10,906 h^−1^) was 42.4‐ and 2.2‐times higher than that of Pt/CeO_2_‐500 (257 h^−1^) and Ru/CeO_2_‐500 (4,982 h^−1^), respectively (Figure 4e). These results clearly demonstrate that the Janus nanostructure not only preserves but also synergistically integrates the distinct functions of Pt and Ru, leading to superior catalytic performance.

Finally, the advantage of each functional specialization of each domain was further examined from theoretical perspective. The relatively downshifted *d*‐band center of Pt optimizes its interaction with H_2_, enabling efficient electron back‐donation into the H‐H σ^*^ antibonding orbital and thereby facilitating H‐H bond cleavage. Consistent with this, DFT calculations (Figure 5a and Figure S19) reveal a lower energy barrier for H_2_ dissociation on Pt(111) (0.03 eV) compared to Ru(0001) (0.22 eV). This enhanced dissociative capacity on Pt is corroborated experimentally by H_2_ temperature‐programmed reduction (H_2_‐TPR) studies. As shown in Figure 5b, the RuPt/CeO_2_‐500 catalysts exhibit a primary H_2_ consumption peak at a significantly lower temperature (∼137°C) than that of the Ru/CeO_2_‐500 counterpart (∼223°C). Collectively, these computational and experimental results confirm the enhanced ability of Pt‐rich domains to activate H_2_ and generate surface ^*^H species.

**FIGURE 5:**
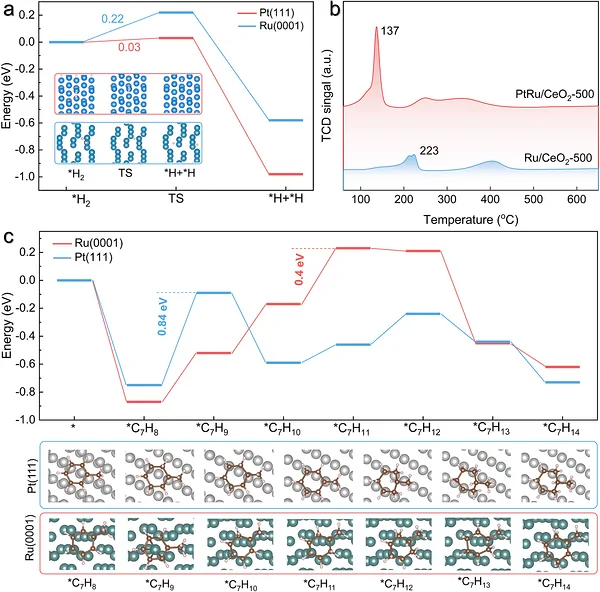
Mechanistic insight into the complementary roles of Pt and Ru active sites. (a) Energy profiles for H_2_ dissociation on Pt(111) and Ru(0001) surfaces. (b) H_2_‐TPR profiles of the PtRu/CeO_2_‐500 and Ru/CeO_2_‐500 catalysts. (c) Free energy profiles of the toluene hydrogenation on Pt(111) and Ru(0001) surfaces.

Conversely, the Ru domains exhibit higher performance in the sequential hydrogenation of toluene. DFT calculations of the reaction free‐energy of intermediates show that toluene adsorbs more strongly on Ru(0001) than that on Pt(111) (Figure 5c and Figure S20). More critically, the most endothermic step on Ru(0001) is the combined ^*^C_7_H_10_ with ^*^H to ^*^C_7_H_11_, which requires only 0.4 eV. In contrast, the corresponding most endothermic step on Pt(111), the formation of ^*^C_7_H_9_, demands 0.84 eV, which is 2.1 times higher than the energy requirement on Ru(0001) (Figure 5c). Furthermore, the final product methylcyclohexane binds more weakly to Ru(0001) compared to Pt(111), favoring its desorption and thereby promoting catalyst regeneration. The lower overall energy pathway and easier product release underscore the strong capacity of Ru‐rich domains to drive the complete hydrogenation of toluene to methylcyclohexane.

Based on the above analysis, we propose a synergistic reaction pathway enabled by the reconstructed Janus PtRu nanostructures in PtRu/CeO_2_‐500 (Figure 1b). H_2_ undergoes facile dissociation on the Pt‐rich domains to generate ^*^H species, while toluene is preferentially adsorbed and activated on the adjacent Ru‐rich regions. The generated ^*^H species subsequently spill over onto the Ru sites, where they hydrogenation the activated toluene. This Janus reconstruction uniquely integrates the complementary functions of Pt and Ru within a single nanostructure, combining the high H_2_ activation capability of Pt with the high intrinsic activity of Ru for toluene hydrogenation. Such a synergistic configuration ultimately drives the exceptional low‐temperature hydrogenation activity observed.

Additionally, this SMSI‐driven Janus reconstruction in PtRu alloys is not limited to the CeO_2_(110) surface. To probe its generality, we prepared CeO_2_ nanocubes (NC‐CeO_2_), which predominantly expose the CeO_2_(100) surface [47, 48]. Following the same impregnation procedure with nominal loadings of Pt (1 wt.%) and Ru (2 wt.%), the PtRu/NC‐CeO_2_ catalyst was reduced at 200°C and 500°C to yield PtRu/NC‐CeO_2_‐200 and PtRu/NC‐CeO_2_‐500, respectively. XPS analysis revealed that PtRu/NC‐CeO_2_‐500 exhibited the comparable Ru electronic properties, but a significantly higher proportion of reduced Pt species compared to PtRu/NC‐CeO_2_‐200 (Figure S21), confirming the successful occurrence of Janus reconstruction. Consequently, PtRu/NC‐CeO_2_‐500 exhibited significantly higher catalytic activity for toluene hydrogenation (Figure S22). Furthermore, this SMSI‐driven Janus reconstruction can also be extended to another reducible oxide support, TiO_2_. XPS analysis confirmed the successful formation of Janus nanostructures on PtRu/TiO_2_ (Figure S23), which likewise resulted in the enhanced catalytic activity of toluene hydrogenation (Figure S24). Nevertheless, these observations do not imply that SMSI‐induced Janus reconstruction is universally applicable to all support surfaces. Rather, it requires two prerequisites: (i) a sufficiently large difference in the metal‐support interactions of the two constituent metals, and (ii) an appropriately small alloy particle size to allow kinetically feasible reconstruction. DFT calculations can serve as a useful tool for preliminary screening of suitable metal‐support combinations.

## 3 Conclusion

This work establishes differential SMSI as a thermodynamic lever for engineering Janus nanoparticles on oxide supports. Using PtRu/CeO_2_ as a proof‐of‐concept system, we show that the marked difference in metal‐support affinity, in which Ru binding strongly to CeO_2_ while Pt interacts weakly, can be harnessed to drive spontaneous phase separation during high‐temperature reduction. This SMSI‐induced reconstruction spontaneously yields an intimate Janus architecture that spatially decouples two complementary functions: Pt‐rich domains serve as highly active sites for H_2_ dissociation, while Ru‐rich domains facilitate toluene adsorption and activation. The seamless interface enables efficient hydrogen spillover, synergistically integrating these capabilities and delivering exceptional low‐temperature activity for toluene hydrogenation (TOF = 10 906 h^−1^ at 50°C). Beyond the specific system, this work establishes differential SMSI as a versatile thermodynamic lever for driving phase separation and organizing multiple active sites within a single nanoparticle. The principle is broadly applicable to other bimetallic combinations and reducible supports, offering a rational and scalable pathway to design advanced multifunctional catalysts. We anticipate that this strategy will inspire the development of efficient catalysts not only for hydrogen storage via LOHCs but also for other complex, multi‐step reactions central to sustainable energy conversion and chemical synthesis.

## 4 Experimental Section

### 4.1 Preparation of the CeO_2_ Nanorods

CeO_2_ nanorods were synthesized via a hydrothermal method [49]. Briefly, an aqueous solution of Ce(NO_3_)_3_ (10 mL, 0.8 mmol mL^−1^) was added dropwise to a NaOH solution (70 mL, 6.4 mmol mL^−1^) under vigorous stirring at room temperature. After stirring for 30 min, the mixture was transferred into a 100 mL Teflon‐lined stainless‐steel autoclave and heated at 100°C for 24 h. The resulting solid product was collected by centrifugation, washed alternately with H_2_O and ethanol three times, and finally dried at 60°C overnight.

### 4.2 Preparation of the PtRu/CeO_2_‐T catalysts

Typically, 300 mg of the as‐prepared CeO_2_ nanorods were dispersed in 30 mL of ethanol. Aqueous solutions of RuCl_3_ (5 mg_Ru_ mL^−1^) and H_2_PtCl_6_ (5 mg_Pt_ mL^−1^) were added to achieve nominal loadings of 2 wt.% Ru and 1 wt.% Pt. The mixture was stirred at room temperature for 2 h and then evaporated at 80 °C. The obtained powder was subsequently reduced under a 5 *vol*.% H_2_/Ar gas flow (20 mL min^−1^). The temperature was ramped at 5 °C min^−1^ and held at T°C for 2 h. The resulting catalysts are denoted as PtRu/CeO_2_‐T, where T represents the reduction temperature (200°C, 350°C, and 500 °C).

### 4.3 Catalytic Hydrogenation of Toluene

The hydrogenation of toluene was performed in a 500 mL stainless‐steel autoclave. Typically, 10 mg of catalyst was dispersed in 9.6 mmol of toluene within a 5 mL quartz bottle reactor. The reactor was transfer into the stainless‐steel autoclave. Then, the system was sealed, purged three times with H_2_ to remove air, and pressurized to 1 MPa pressure. The reaction was conducted at a set temperature (30°C –60°C) with stirring at 600 rpm. After the reaction, the autoclave was cooled to room temperature in an ice‐water bath. The liquid products were analyzed by gas chromatography (GC). The detailed analysis process can be found in the Supplementary Materials (Figures S25 and S26).

The reaction rate (*r*) was calculated by the following equation:

$$r\;=\frac{n_{0}\times C}{t}$$

The turn over frequency (TOF) values were calculated by the following equation:

$$\mathrm{TOF}\;=\frac{n_{0}\times C}{t\times n_{cat}\times D}$$

where *n_0_* is the moles mass of toluene, mmol; *C* is the conversion of reactants at the specific reaction time of *t*, %; *n_cat_* is the molar mass of Ru atoms, mmol; *t* is the reaction time, h; *D* is the dispersion of Ru atoms, %.

## Author Contributions

**Xingyu Zhang**: data curation, formal analysis, Writing – original draft, methodology. **Yunhua Xu**: supervision, resources, funding acquisition. **Haiqiang Bai**: data curation. **Wenbin Li**: data curation, formal analysis. **Sai Zhang**: methodology, investigation, writing – original draft, writing – review and editing. **You Wang**: methodology, data curation.

## Conflicts of Interest

The authors declare no conflicts of interest.

## Supporting information




**Supporting File**: advs77432‐sup‐0001‐SuppMat.docx.

## Data Availability

The data that support the findings of this study are available from the corresponding author upon reasonable request.
