# Supplementary material for: Strong Metal‐Support Interaction Driven Janus Reconstruction in PtRu Alloys for Low‐Temperature Hydrogen Storage
Source: Adv Sci (Weinh). 2026 Aug 27:e77432. Online ahead of print. doi: 10.1002/advs.77432 (PMC13521184; doi:10.1002/advs.77432)
Supplement: Supplementary file 1 [file ADVS-9999-e77432-s001.docx]

**Supporting information**

**Strong Metal-Support Interaction Driven Janus Reconstruction in PtRu Alloys for Low-temperature Hydrogen Storage**

Xingyu Zhang,^#,[a,b]^ Haiqiang Bai,^#,[b]^ Wenbin Li,^[c]^ You Wang,^[c]^ Yunhua Xu,^[b]^ and Sai Zhang,* ^[c]^

[a]	X. Y. Zhang, H. Q. Bai, Prof. Y. H. Xu
College of New Energy, Yulin University, Yulin 719000, China

[b]	X. Y. Zhang
School of Material Science and Engineering, Xi'an University of Technology, Xi'an 710048, China

[c]	W. B. Li, Y. Y. Wang, Prof. S. Zhang
School of Chemistry and Chemical Engineering, Northwestern Polytechnical University, Xi'an 710048, China
E-mail: [zhangsai1112@nwpu.edu.cn](mailto:zhangsai1112@nwpu.edu.cn)

[^#^]	These authors contributed equally.

**Materials**

Cerium nitrate hexahydrate (Ce(NO_3_)_3_^.^6H_2_O), sodium hydroxide (NaOH), ruthenium chloride hydrate (RuCl_3_·xH_2_O) and chloroplatinic acid hydrate (H_2_PtCl_6_·6H_2_O) were obtained from Energy Chemical. All reagents were of analytical grade and were used as received without further purification prior to the experiments

**Characterizations**

Transmission electron microscopy (TEM) was performed on a Hitachi HT-7700 operating at 120 kV. X-ray photoelectron spectroscopy (XPS) spectra were acquired on a Thermo Scientific K-Alpha spectrometer with Al Kα excitation. X-ray diffraction (XRD) patterns were recorded on a Bruker D8 Discover diffractometer using Cu Kα radiation. H_2_ temperature-programmed reduction (H_2_-TPR) was carried out on an AutoChem II 2920 instrument. Diffuse reflectance infrared Fourier transform spectroscopy (DRIFTS) was conducted on a Bruker Vertex 70 spectrometer equipped with an *in situ* reaction cell.

**Diffuse reflectance infrared Fourier transforms spectroscopy (DRIFTS)**

DRIFTS analysis was conducted using a Bruker INVENIO S spectrometer equipped with a liquid nitrogen-cooled MCT detector. Prior to measurements, samples were pretreated under 30 mL min^-1^ Ar flow at 150 °C for 30 min. Following cooling to 30 °C, by flowing D_2_ (30 mL min^-1^) at ambient temperature for 30 min adsorption. Subsequently, physisorbed deuterium gas was removed via purging under 30 mL min^-1^ N_2_ flow (10 min). Time-resolved desorption profiles were acquired by collecting spectra at 5-min intervals under continuous N_2_ flow (30 mL min^-1^).

**DFT calculations**

Periodic spin-polarized DFT calculations were performed using the Vienna Ab initio Simulation Package (VASP, version 6.3.0) within the generalized gradient approximation (GGA) as parameterized by Perdew-Burke-Ernzerhof (PBE). The projector augmented wave (PAW) method was employed to describe the core-electron interactions for Ce (4f, 5s, 5p, 5d, 6s) and O (2s, 2p). Kohn-Sham valence states were expanded in a plane-wave basis set with a cutoff energy of 400 eV. Spin polarization was enabled (ISPIN = 2). Van der Waals interactions were accounted for using the DFT‑D3(BJ) correction.

The equilibrium lattice constant of bulk CeO_2_ (5.49 Å) was determined at the PBE+U level (U = 4.5 eV) to be 5.49 Å. A CeO_2_(110) surface slab model was constructed from a (4×4) supercell containing three Ce-O-Ce tri-layers (Ce_48_O_96_). A vacuum gap of 15 Å was introduced to avoid periodic interactions. Climbing-image nudged elastic band (CI-NEB) calculations were performed using the same settings as for geometry optimizations. The energy convergence criterion was set to 10^-4^ eV, and the maximum force allowed on each atom during structural relaxation was 0.05 eV Å^-1^.

The adsorption energy (*E_ads_*) was defined as: *E_ads_* = *E_total_* – *E_slab_* − *E_adsorbate_*, where *E_total_* and *E_slab_* are the total energies of a surface with adsorbates and a clean surface, respectively. *E_adsorbate_* is the total energy of the free adsorbate. The d‑band center was calculated from the projected density of states obtained with the same computational setup.

**Figure S1. Optimized adsorption configuration.** Optimized adsorption configurations of (a) Pt_1_ and (b) Ru_1_ atom on the CeO_2_(110) surface (top and side views).

**Figure S2. Optimized adsorption configuration.** Optimized adsorption configurations of (a) Pt_10_ and (b) Ru_10_ cluster on the CeO_2_(110) surface (top and side views).

**Figure S3. Characterization of the CeO_2_ supports.** (a) XRD pattern and (b) (c) (d) HAADF-STEM images of the CeO_2_ supports.

**Figure S4.** XRD patterns. XRD patterns of the PtRu/CeO_2_-200, PtRu/CeO_2_-350 and PtRu/CeO_2_-500 catalysts.

**Figure S5. Characterization of the PtRu/CeO_2_-200 catalyst.** (a) HAADF-STEM image of PtRu/CeO_2_-200. (b) Size distribution of PtRu nanoparticles for PtRu/CeO_2_-200.

**Figure S6. Characterization of the PtRu/CeO_2_-500 catalyst.** (a) HAADF-STEM image of PtRu/CeO_2_-500. (b) Size distribution of PtRu nanoparticles for PtRu/CeO_2_-500.

**Figure S7. XPS analysis.** XPS analysis of (a) Ce 3*d* and (b) O 1*s* peak for the PtRu/CeO_2_-T catalysts. **Note:** The Ce^4+^ species are represented by the spin-orbit doublet components [v, v’’, v’’’] and [u, u’’, u’’’], where v, v’’, and v’’’ correspond to the Ce 3*d_5/2_* level, while u, u’’, and u’’’ correspond to the Ce 3*d_3/2_* level. The Ce^3+^ species are represented by the components [v_0_, v’] and [u_0_, u’], where v_0_ and v’ correspond to the Ce 3*d_5/2_* level, and u_0_ and u’ correspond to the Ce 3*d_3/2_* level. The Ce^3+^ fraction was calculated by the S(v_0_ + v’)/S(v_0_ + v + v’ + v’’ + v’’’). The binding energies at ~529.2 eV, ~530.6 eV and ~532.3 eV are attributed to lattice oxygen (Ce^4+^-O), defect-related oxygen species (Ce^3+^-O) and hydroxyl groups (-OH) or adsorbed water on the CeO_2_ surface. The Ce^3+^-O fraction was calculated by the S(Ce^3+^-O)/S(Ce^4+^-O + Ce^3+^-O + -OH).

**Figure S8. *K*-edge EXAFS spectra.** *K*-edge EXAFS spectral data of Ru elements in (a) PtRu/CeO_2_-200, (b) PtRu/CeO_2_-350 and (c) PtRu/CeO_2_-500, derived from *k^3^* weighted Fourier transform spectra.

**Figure S9. *L*-edge EXAFS spectra.** *L*-edge EXAFS spectral data of Pt elements in (a) PtRu/CeO_2_-200, (b) PtRu/CeO_2_-350 and (c) PtRu/CeO_2_-500, derived from *k^3^* weighted Fourier transform spectra.

**Figure S10. Catalytic performance of PtRu/CeO_2_ for toluene hydrogenation.** The influence of mass ratio of Ru:Pt on toluene hydrogenation. **Reaction conditions:** catalyst (10 mg), toluene (1 mL), 50 °C, 1 MPa H_2_ and 2 h.

**Figure S11. Kinetic data.** (a) Time-dependent toluene conversion over PtRu/CeO_2_-500 during toluene hydrogenation. (b) correlation between ln *r* and 1/*T* for PtRu/CeO_2_-200, PtRu/CeO_2_-350 and PtRu/CeO_2_-500. **Reaction conditions:** catalyst (10 mg), toluene (1 mL), 50 °C, 1 MPa H_2_.

**Figure S12. Characterization of the spent PtRu/CeO_2_-500 catalyst.** (a) Dark-field TEM image and (b) size distribution of PtRu nanoparticles for the spent PtRu/CeO_2_-500 catalyst.

**Figure S13. Characterization of the spent PtRu/CeO_2_-500 catalyst.** (a) XRD patterns. (b) Ru 3*p* and (c) Pt 4*f* core-level spectra of PtRu/CeO_2_-500 and spent PtRu/CeO_2_-500.

**Figure S14. HAADF-STEM image of PtRu/CeO_2_-200.**

**Figure S15. Characterization of the Pt/CeO_2_-500 catalyst.** (a) Dark-field TEM image and (b) size distribution of Pt nanoparticles for the Pt/CeO_2_-500 catalyst.

**Figure S16.** **Characterization of the Ru/CeO_2_-500 catalyst.** (a) Dark-field TEM image and (b) size distribution of Ru nanoparticles for the Ru/CeO_2_-500 catalyst.

**Figure S17. XPS spectra of Ru/CeO_2_-500 and Pt/CeO_2_-500.** (a) Ru 3*p* and (d) Pt 4*f* core-level spectra of Ru/CeO_2_-500 and Pt/CeO_2_-500.

**Figure S18. Catalytic performance of toluene hydrogenation.** Toluene conversion over the Ru/CeO_2_-500, Pt/CeO_2_-500 and PtRu/CeO_2_-500 catalysts. **Reaction conditions:** catalyst (5 mg), toluene (1 mL), 50 °C and 1 MPa H_2_.

**Figure S19. Optimized H_2_ dissociation configuration.** Optimized dissociation configuration of H_2_ on the Pt(111) and Ru(0001) surfaces.

**Figure S20. Optimized configuration of the toluene hydrogenation.** Optimized configuration of the toluene hydrogenation on Pt(111) and Ru(0001) surfaces.

**Figure S21. XPS spectra of the PtRu/NC-CeO_2_ catalysts.** (a) Ru 3*p* and (d) Pt 4*f* core-level spectra of PtRu/NC-CeO_2_-200 and PtRu/NC-CeO_2_-500.

**Figure S22. Catalytic performance of PtRu/NC-CeO_2_-200 and PtRu/NC-CeO_2_-500 for toluene hydrogenation.** **Reaction conditions:** catalysts (10 mg), toluene (1 mL), 50 °C, 1 MPa H_2_ and 2 h.

**Figure S23. XPS spectra of the PtRu/TiO_2_ catalysts.** (a) Ru 3*p* and (d) Pt 4*f* core-level spectra of PtRu/TiO_2_-200 and PtRu/TiO_2_-500.

**Figure S24. Catalytic performance of PtRu/NC-CeO_2_-200 and PtRu/NC-CeO_2_-500 for toluene hydrogenation.** **Reaction conditions:** catalysts (10 mg), toluene (1 mL), 50 °C, 1 MPa H_2_ and 2 h.

**Figure S25.** **Calibration curves.** Standard correlations of GC integral area versus concentration for (a) toluene and (b) methylcyclohexane.

**Figure S26. Typical GC spectra.** Typical GC spectra of toluene hydrogenation to methylcyclohexane catalyzed by PtRu/CeO_2_-X.

**Table S1.** Summary of the electronic properties of Pt for PtRu/CeO_2_-T

| Catalysts | Area | | | | | | Pt^0^ fractions  (%) | Pt^2+^ fractions  (%) | Pt^4+^ fractions  (%) |
| --- | --- | --- | --- | --- | --- | --- | --- | --- | --- |
|  | Pt 4*f*_7/2_ | | | Pt 4*f*_5/2_ | | |  |  |  |
|  | Pt^0^ | Pt^2+^ | Pt^4+^ | Pt^0^ | Pt^2+^ | Pt^4+^ |  |  |  |
| PtRu/CeO_2_-200 | 71.79 | 72.86 | 74.00 | 75.1 | 76.16 | 77.56 | 19.0 | 58.2 | 22.8 |
| PtRu/CeO_2_-350 | 71.71 | 72.70 | 74.39 | 75.49 | 76.00 | 77.69 | 22.1 | 57.9 | 20.0 |
| PtRu/CeO_2_-500 | 71.89 | 72.88 | 73.75 | 75.19 | 76.18 | 77.05 | 29.4 | 52.1 | 18.5 |
| Spent PtRu/CeO_2_-500 | 71.93 | 72.82 | 74.16 | 75.13 | 76.12 | 77.46 | 26.1 | 54.5 | 19.4 |
| Pt/CeO_2_-500 | 71.74 | 72.3 | 73.88 | 75.04 | 75.6 | 77.18 | 38.9 | 49.7 | 11.4 |

**Table S2.** Summary of the electronic properties of Ru for Pt/Ru/CeO_2_-T

| Catalysts | Area | | | | | | Ru^0^ fraction  (%) | Ru^2+^ fraction  (%) | Ru^4+^ fraction  (%) |
| --- | --- | --- | --- | --- | --- | --- | --- | --- | --- |
|  | Ru 3*p_3/2_* | | | Ru 3*p_1/2_* | | |  |  |  |
|  | Ru^0^ | Ru^2+^ | Ru^4+^ | Ru^0^ | Ru^2+^ | Ru^4+^ |  |  |  |
| PtRu/CeO_2_-200 | 461.3 | 462.33 | 465 | 483.87 | 484.74 | 487.17 | 23.6 | 52.8 | 23.6 |
| PtRu/CeO_2_-350 | 461.56 | 462.65 | 466.25 | 483.96 | 485.08 | 488.65 | 20.1 | 58.4 | 21.5 |
| PtRu/CeO_2_-500 | 461.29 | 462.86 | 465.7 | 483.04 | 484.8 | 486.73 | 19.2 | 60.8 | 20.0 |
| Spent PtRu/CeO_2_-500 | 461.41 | 463 | 466.31 | 483.81 | 485.4 | 488.8 | 17.9 | 67.6 | 14.4 |
| Ru/CeO_2_-500 | 461.24 | 462.7 | 466.83 | 483.64 | 485.1 | 489.23 | 18.3 | 64.3 | 17.4 |

**Table S3.** Structure parameters and estimated uncertainties (in parentheses) from EXAFS analysis of the PtRu/CeO_2_-200, PtRu/CeO_2_-350 and PtRu/CeO_2_-500 catalysts

| **Sample** | **Paths** | ***N*** | ***R* (Å)** | **σ^2^ (×10^-3^ Å^2^)** | **Δ*E_0_* (eV)** | ***R* factor** |
| --- | --- | --- | --- | --- | --- | --- |
| RuO_2_ | Ru-O | 6 | 1.98 | - | - | - |
| Ru foil | Ru-Ru | 6 | 2.65 | - | - | - |
| PtRu foil | Ru-Pt | 8 | 2.76 | - | - | - |
| PtRu/CeO_2_-200 | Ru-O | 3.74 ± 0.7 | 1.99 ± 0.02 | 9.0 ± 1.9 | 3.75 ± 0.6 | 0.012 |
|  | Ru-Ru | 2.37 ± 0.8 | 2.67 ± 0.03 | 2.1 ± 1.5 |  |  |
|  | Ru-Pt | 3.13 ± 1.3 | 2.76 ± 0.06 | 2.6 ± 1.5 |  |  |
| PtRu/CeO_2_-350 | Ru-O | 3.43 ± 0.5 | 1.94 ± 0.02 | 8.6 ± 3.0 | 4.65 ± 0.6 | 0.015 |
|  | Ru-Ru | 3.36 ± 0.5 | 2.67 ± 0.01 | 4.6 ± 0.8 |  |  |
|  | Ru-Pt | 1.62 ± 0.8 | 2.76 ± 0.01 | 9.4 ± 0.4 |  |  |
| PtRu/CeO_2_-500 | Ru-O | 3.36 ± 0.5 | 1.97 ± 0.03 | 8.7 ± 2.9 | 6.50 ± 1.8 | 0.019 |
|  | Ru-Ru | 3.93 ± 0.8 | 2.69 ± 0.01 | 4.6 ± 0.7 |  |  |
|  | Ru-Pt | 0.23 ± 0.6 | 2.76 ± 0.03 | 5.6 ± 3.8 |  |  |
| PtO_2_ | Pt-O | 8 | 2.07 | - | - | - |
| Pt foil | Pt-Pt | 12 | 2.76 | - | - | - |
| PtRu foil | Ru-Pt | 8 | 2.75 | - | - | - |
| PtRu/CeO_2_-200 | Pt-O | 4.45 ± 0.7 | 2.04 ± 0.05 | 8.5 ± 1.5 | 4.6 ± 0.2 | 0.015 |
|  | Pt-Pt/Ru | 2.28 ± 1.3 | 2.77 ± 0.05 | 10.6 ± 2.8 |  |  |
| PtRu/CeO_2_-350 | Pt-O | 3.23 ± 0.7 | 2.04 ± 0.04 | 8.8 ± 3.4 | 9.4 ± 1.8 | 0.016 |
|  | Pt-Pt/Ru | 3.82 ± 0.6 | 2.77 ± 0.03 | 7.4 ± 0.5 |  |  |
| PtRu/CeO_2_-500 | Pt-O | 1.71 ± 0.4 | 2.04 ± 0.01 | 4.8 ± 2.4 | 11.4 ± 1.3 | 0.015 |
|  | Pt-Pt/Ru | 5.55 ± 0.2 | 2.77 ± 0.03 | 10.7 ± 1.7 |  |  |

**Table S4.** Summary of the catalytic performance of toluene hydrogenation

| **Entry** | **Catalysts** | **T**  **(°C )** | **P**  **(MPa)** | **Time**  **(h)** | **Conv.**  **(%)** | **TOF**  **(h^-1^)** | **Ref.** |
| --- | --- | --- | --- | --- | --- | --- | --- |
| 1 | PtRu/CeO_2_ | 50 | 1 | 2 | 73 | 10906 | This work |
| 2 | PtRh@HMSNs | 30 | 0.1 | 2 | 100 | 99 | S1 |
| 3 | Ru@C@YSN-500 | 50 | 2 | 2 | 99.9 | 5,270 | S2 |
| 4 | Ru-MWCNT | 50 | 4 | 14 | 100 | 100 | S3 |
| 5 | Ru/MgO | 120 | 1 | 1.5 | 100 | 1,100 | S4 |
| 6 | Ru/PVPy | 120 | 1 | 1 | 82 | 69 | S5 |
| 7 | Pt_0.035_Co_5.3_/r-TiO_2_ | 125 | 0.1 | 1.2 | 30 | 83 | S6 |
| 8 | PdRu/SiO_2_ | 130 | 2 | - | 60 | 90 | S7 |
| 9 | Ru/SiO_2_ | 130 | 2 | - | 79 | 1,073 | S8 |
| 10 | Ru/rp-Al_2_O_3_ | 80 | 3 | 3 | 100 | 735 | S9 |
| 11 | Pd/SiO_2_(AERO) | 70 | 1 | 1 | - | 15 | S10 |
| 12 | Pt /Al_2_O_3_-F | 80 | 2 | 12 | 100 | 6993 | S11 |
| 13 | Pt/CeO-A-400 | 100 | 0.5 | 3 | 90.8 | 2,164 | S12 |
| 14 | Run/ND@G | 100 | 1.5 | 3 | 100 | 9580.3 | S13 |
| 15 | Ru/NFPC | 100 | 1.4 | 1 | 63 | 8,752 | S14 |
| 16 | Ru(0)/SiO_2_ | 100 | 3 | 1 | - | 360 | S15 |

**Reference:**

[S1] K. Li, H. An, P. Yan, Hydrogenation of toluene to methyl cyclohexane over PtRh bimetallic nanoparticle-encaged hollow mesoporous silica catalytic nanoreactors, ACS Omega., 6 (2021) 5846-5855.

[S2] M. Guo, G. Lan, Peng J. Enhancing the catalytic activity of Ru NPs deposited with carbon species in yolk–shell nanostructures, J. Mater. Chem. A., 4 (2016) 10956-10963.

[S3] J. Mohamad, K. Yolande, T. Emmanuelle, Montserrat G. Ruthenium nanoparticles supported on multi-walled carbon nanotubes: Highly effective catalytic system for hydrogenation processes, J. Mol. Catal. A-Chem., 332 (2010) 106-112.

[S4] K. Moromi, H. Siddiki, K. Kon, Acceptorless dehydrogenation of N-heterocycles by supported Pt. Catal. Today., 281 (2017) 507-511.

[S5] C. Bianchini, V. Dal Santo, A. Meli, S. Moneti, M. Moreno, W. Oberhauser, R. Psaro, L. Sordelli, F. Vizza, Hydrogenation of arenes and N-heteroaromatic compounds over ruthenium nanoparticles on poly(4-vinylpyridine): a versatile catalyst operating by a substrate-dependent dual site mechanism, J. Catal., 213 (2003) 47-62.

[S6] C. Bianchini, V. Dal Santo, A. Meli, S. Moneti, M. Moreno, W. Oberhauser, R. Psaro, L. Sordelli, F. Vizza, A comparison between silica-immobilized ruthenium(II) single sites and silica-supported ruthenium nanoparticles in the catalytic hydrogenation of model hetero- and polyaromatics contained in raw oil materials, J. Catal., 213 (2003) 47-62.

[S7] S. Zhao, J. Cai, Hou Q. Microcalorimetric adsorption and infrared spectroscopic studies of supported Pd, Ru and Pd–Ru catalysts for the hydrogenation of aromatic rings with carboxyl groups. Catal. Sci. Technol., 11 (2021) 3070-3083.

[S8] S. Zhao, J. Cai, Hou Q. Microcalorimetric adsorption and infrared spectroscopic studies of supported Pd, Ru and Pd–Ru catalysts for the hydrogenation of aromatic rings with carboxyl groups. Catal. Sci. Technol., 11 (2021) 3070-3083.

[S9] N. Tang, Y. Cong, Q. Shang, Coordinatively unsaturated Al3+ sites anchored subnanometric ruthenium catalyst for hydrogenation of aromatics, ACS Catal., 7 (2017) 5987-5991.

[S10] Y. Yang, X. Lin, J. Tang, J. Zhang, C. Liu, J. Huang, Supported mesoporous Pt catalysts with excellent performance for toluene hydrogenation under low reaction pressure. Molecular Catalysis., 524 (2022) 112341.

[S11] X. Zhang, Y. K. Xu, Q. K. Liao, Y. Sun, S. Zhang, Regulating the d-band center of Pt for highly effective H2 storage through toluene hydrogenation at low temperatures, J. Mater. Chem. A., 12 (2024) 23392-23397

[S12] Y. Yang, X. Lin, J. Tang, J. Zhang, C. Liu, J. Huang, Supported mesoporous Pt catalysts with excellent performance for toluene hydrogenation under low reaction pressure. Molecular Catalysis., 524 (2022) 112341.

[S13] Y. Wang, H. Yu, Y. He, S. Xiang, X. Qin, L. Yang, J. Chen, Y. Si, J. Zhang, J. Diao, N. Wang, M. Peng, D. Ma, H. Liu, Angew. Chem. Int. Ed., 64 (2025) e202415542.

[S14] M. Li, J. Tang, M. Deng Nitrogen-doped flower-like porous carbon materials directed by in situ hydrolysed MgO: Promising support for Ru nanoparticles in catalytic hydrogenations, Nano Res., 9 (2016) 3129-3140.

[S15] C. Bianchini, V. Dal Santo, A. Meli, S. Moneti, M. Moreno, W. Oberhauser, R. Psaro, L. Sordelli, F. Vizza, A comparison between silica-immobilized ruthenium(II) single sites and silica-supported ruthenium nanoparticles in the catalytic hydrogenation of model hetero- and polyaromatics contained in raw oil materials, J. Catal., 213 (2003) 47-62.
